# Bone Health in Paediatric Inflammatory Bowel Disease

**DOI:** 10.3390/diagnostics15050580

**Published:** 2025-02-27

**Authors:** Proteek Sen, Suma Uday

**Affiliations:** 1 Department of Endocrinology and Diabetes, Birmingham Women’s and Children’s NHS Foundation Trust, Birmingham B4 6NH, UK; proteek.sen1@nhs.net; 2 Department of Metabolism and Systems Science, College of Medical and Dental Sciences, University of Birmingham, Birmingham B15 2TT, UK

**Keywords:** systemic chronic inflammation, biomarkers of inflammation, imaging in inflammatory pathology, osteoporosis, fractures, DXA, nutrition, vitamin D, bisphosphonates

## Abstract

Paediatric inflammatory bowel disease (IBD) is often complicated by bone loss resulting in an increased risk of fractures and impaired quality of life. Underlying inflammation, nutritional deficiencies and glucocorticoid therapy are some of the factors contributing to secondary osteoporosis in IBD. Optimising nutrition, dietary supplementation and timely screening are essential in preventing bone loss. Bisphosphonate therapy remains the cornerstone of medical management of osteoporosis. This review explores the various mechanisms contributing towards poor bone health in IBD and the recent advances in diagnostic and preventive approaches along with updates in management strategies.

## 1. Introduction

Inflammatory bowel disease (IBD) is a chronic, relapsing remitting inflammatory condition which includes two major subtypes: ulcerative colitis (UC) and Crohn’s disease (CD) [1]. In 2017, there were 6.8 million cases of IBD globally [2]. There has been a significant rise in the incidence of paediatric IBD in the past decade, notably in south Asia and India [3,4,5]. IBD is generally considered to be a systemic disease with various extraintestinal manifestations [6]. Osteoporosis is an important extraintestinal manifestation of paediatric IBD and the causes are multifactorial [7,8]. Patients with IBD are at a significantly higher risk for low bone mineral density (BMD), especially in CD [9,10]. Bone-mass accrual in childhood is an important determinant of skeletal health in the later stages of life, and adequate bone mass after adolescence is protective in adulthood [11]. Low bone density in children and adolescents with IBD can lead to decreased skeletal strength and increased risk of fractures [12,13,14]. In this review we delve deeper into the potential mechanisms contributing to bone deficits and discuss the prevention and management strategies to improve bone health in children with IBD.

## 2. Bone Health in IBD

IBD can have a significant impact on patients’ linear growth, pubertal development, nutritional status and bone health [15]. Dubner et al. demonstrated substantial musculo-skeletal deficits in children with CD at baseline, attributable to the underlying inflammation and malnutrition [16]. Additional factors which contribute towards impaired bone health in IBD include vitamin malabsorption, lack of physical activity and prolonged glucocorticoid therapy [17].

## 3. Osteoporosis and Fracture Risk in IBD

Children with IBD are more prone to osteoporosis than the general population [18]. BMD assessed by dual-energy X-ray absorptiometry (DXA) is frequently used in the evaluation of bone health and gives an estimation of fracture risk. BMD is one of the most widely used measures in the diagnosis of osteoporosis [19]. Low bone mineral density is the preferred term for paediatric DXA reports when areal BMD Z-scores are less than or equal to −2.0 SD [20]. Paediatric osteoporosis is defined by the combination of a BMD Z-score ≤−2 AND a clinically significant fracture (>2 long-bone fractures before 10 years of age or >3 long-bone fractures up to 19 years of age) OR > 1 vertebral compression fractures occurring without high-energy trauma or local disease irrespective of the BMD Z-score [20]. As DXA is a two-dimensional method, BMD may be interpreted incorrectly due to smaller bones in children, especially in chronic diseases [21]. This is corrected with estimation of BMAD (bone mineral apparent density), which adjusts the size-related effects on BMD results by deriving a three-dimensional bone volume from the two-dimensional bone area provided by DXA [22]. Hence, children with short stature may have an abnormal BMD and a normal BMAD [23].

Peripheral quantitative computed tomography (pQCT) studies provide three-dimensional assessments of bone density (volumetric BMD, vBMD) as opposed to two-dimensional measures provided by DXA [24]. pQCT is also useful in distinguishing between cortical and trabecular bone [24]. The radiation exposure from pQCT is low and only slightly higher when compared to DXA [25]. The use of pQCT is limited largely to research settings due to the paucity of reference data, variability in scanning protocols and its inability to assess whole body parameters [26,27].

A study of over 737 cases of CD found young children (<12 years) to be at a higher risk and to have a higher prevalence of fractures compared to non-IBD controls [28]. In a systematic review and meta-analysis of adult patients with IBD compared to healthy controls, individuals with IBD were found to have a 38% greater risk of fractures and to be at a significantly higher risk for vertebral fractures [29]. Vertebral fracture risk in adults is recorded at 22% [30]. Vertebral fracture data in children are limited [31]. One cross-sectional study of 216 children with very early onset IBD (onset before 6 years) reported vertebral fractures in 1.4% of the cohort [32].

Increased disease activity in IBD has been shown to have a negative effect on bone microarchitecture [24]. In a study of 102 young patients (12–33 years old) with IBD, Pepe et al. demonstrated decreased trabecular vBMD and alterations in trabecular and cortical bone microarchitecture in IBD patients compared to age-, sex- and height-matched controls [24].

Disorders of mineral metabolism, frequently reduced mineral absorption secondary to severe malabsorption, can be seen in IBD. Vitamin D and/or calcium deficiency can result in rickets and/or osteomalacia and is characterised by the accumulation of undermineralised bone, which can manifest as bone deformities, skeletal pain or proximal muscle weakness [33]. Along with typical biochemical features (see later), rickets is definitively diagnosed on radiographs and osteomalacia on bone biopsies; DXA scans do not play a role in the diagnosis of rickets/osteomalacia [34]. However, it is important to note that osteomalacia may co-exist with osteoporosis [35].

## 4. Pathophysiology of Low Bone Mass in Paediatric IBD

### Bone Remodelling, Modelling and the Impact of Inflammation

Bone remodelling is a dynamic process in which old and damaged bone is continuously replaced by new bone, and it is essential for preserving skeletal integrity [36,37]. Bone formation (osteoblast-mediated) and resorption (osteoclast-mediated) is a tightly regulated process [36]. The coupling of the two processes by multiple coordinated signals is essential for optimum bone mineralisation [36]. The carefully coordinated interplay between osteoblasts and osteoclasts plays a major role in bone remodelling [38].

Osteoblasts are bone-forming cells that originate from mesenchymal stem cells [39]. Osteoclasts, which arise from haematopoietic progenitors, are multinucleated cells that break down bone tissue by forming an acid compartment and releasing proteases, called tartrate-resistant acid phosphatase that degrades both inorganic and bone components [39,40]. Osteoclast progenitor cells are chemotactically attracted to sites of bone resorption, where they deposit, proliferate and finally differentiate into osteoclasts [41]. Osteocytes are cells that control osteoclast and osteoblast activity and hence regulate bone remodelling [42]. The interplay between these cells is largely mediated by the osteoprotegerin (OPG) and receptor activator of the NF-κB system ligand (RANKL) system [43]. RANK, its ligand RANKL and OPG belong to the tumour necrosis factor (TNF) and its receptor superfamilies [43]. RANKL is present on the surface membrane of osteoblasts [44]. RANKL binds to RANK receptors on the surface of osteoclasts and their precursors, facilitating the maturation of osteoclasts [45]. OPG behaves as a decoy soluble receptor for RANKL and inhibits osteoclast activity by preventing RANKL from binding to RANK, thereby inhibiting osteoclast formation [46]. The differential influence of RANKL and OPG regulate bone homoeostasis and determine net bone formation or resorption [47]. Disruption of the RANKL–RANK–OPG axis leads to the uncoupling of bone metabolism [48]. Pro-inflammatory cytokines, such as interleukin (IL)-1, IL-6, IL-17 and especially TNF-α induce and enhance RANKL expression, increasing the ratio of RANKL to OPG, enhancing osteoclastogenesis and eventually bone resorption [49,50].

Paediatric bones elongate and alter their shape by a process called bone modelling, which is different from bone remodelling and occurs almost exclusively in children [51]. In bone modelling, both osteoblasts and osteoclasts are active simultaneously but on different parts of the bone [51]. Chronic inflammatory diseases, such as IBD, have an inhibitory effect on bone modelling and hence linear growth [52].

## 5. Causes of Low Bone Mass in Children with IBD

The causes of poor bone health in children with IBD are multidimensional, ranging from non-modifiable factors such as age and gender to modifiable factors such as nutrition and physical activity [53] and disease and treatment related factors such as disease severity or steroid use.

### 5.1. Non-Modifiable Factors

Gender-based differences have been observed in bone metabolism in children with IBD but with varying results [53]. Gokhale et al. conducted a study including 99 children with IBD and found that pubertal and postpubertal girls were most likely to have low bone mass [8]. However, the male gender has also been noted to be a risk factor for low BMD [54].

### 5.2. Disease- and Treatment-Related Factors

#### 5.2.1. Type of IBD

Individuals with CD, when compared to those with UC, have been shown to have an increased predisposition towards negative effects on bone microarchitecture, including lower trabecular mineral density and lower cortical thickness [53,55,56].

#### 5.2.2. Role of Low Muscle Mass

The mechanostat theory dictates that an increase in muscle mass and strength results in an increase in bone mass and bone strength [57]. Underlying inflammation and glucocorticoid use increase the expression of myostatin that inhibits skeletal muscle differentiation [58,59]. Ward et al. demonstrated low muscle and bone mass at multiple analysed sites in newly diagnosed paediatric patients with CD [60]. Dynamic muscle function testing also demonstrated muscle-strength impairment [60].

#### 5.2.3. Role of Corticosteroids

There are various osteotoxic effects of glucocorticoids on bone and mineral metabolism that may cause osteoporosis [61]. Glucocorticoids increase the apoptosis of osteoblasts and mature osteocytes via activation of caspase 3 [62]. Glucocorticoid use increases reactive oxygen species production, resulting in suppression of the Wnt/β-catenin pathway, which is necessary for osteoblastogenesis [63]. Glucocorticoids stimulate osteoclastogenesis by profoundly inhibiting OPG and subsequently stimulating RANKL expression, leading to a hyper-resorptive state [64,65]. Low-dose glucocorticoids cause osteocyte autophagy and high-dose or prolonged usage may cause osteocyte necrosis and apoptosis [66].

Proteoglycans play an important role in chondrogenesis and cell proliferation during early embryonic development [67]. Rat studies have shown that dexamethasone inhibits the activity of uridine diphosphate glucose dehydrogenase, a key enzyme in the synthesis of proteoglycans [68]. Dexamethasone has also been shown to cause chondrocyte apoptosis [69].

Vihinen et al. found that bone formation markers such as amino-terminal type I collagen propeptide (PINP) levels were significantly lower in patients with active IBD before treatment as compared with IBD controls in clinical remission, which could be attributed to the negative effect of inflammatory cytokines on bone formation [70]. Glucocorticoid therapy initiation further reduced PINP levels [70].

#### 5.2.4. Role of Poor Linear Growth and Delayed Puberty

A quarter of patients with IBD are diagnosed in childhood and the majority during pubertal years [71,72]. A systematic review of 41 studies across the USA, Canada and Europe, evaluating 3505 CD patients, 2071 UC patients, and 461 indeterminate colitis patients younger than 18 years, reported the incidence of growth failure at diagnosis of CD to be between 10% and 56% and in UC to be between 0 and 10% [73]. Growth hormone, released from the anterior pituitary gland, stimulates insulin-like growth factor-1 (IGF-1) production by the liver, which is fundamental in skeletal growth [74]. IGF-1 also has a protective role against low bone mass and fractures [75]. In IBD there is a generalised reduction in IGF-1 levels due to decreased hepatic production secondary to ongoing inflammatory processes and chronic glucocorticoid use [76,77]. IGF-1 further binds to IGF binding protein-3 (IGFBP-3), whose levels are less affected by undernutrition (as opposed to IGF-1) [78]. IGFBP-3 circulating levels are reported to decrease during active phases of CD and return to normal during remission [79]. Pro-inflammatory cytokines, such as TNF-α, inhibit sex-hormone production by acting either at the pituitary or the gonadal level [80,81].

Gonadal and adrenal androgens play a major role in pubertal bone-mass accrual [82]. Gender dimorphism in bone-mass accrual expressed during puberty with a longer period of bone maturation in males than in females leads to a larger increase in bone size and cortical thickness [83,84]. Bone mass acquired during puberty is a major contributor to peak bone mass, thereby determining osteoporosis and fracture risk in later life [85].

### 5.3. Modifiable Factors

#### 5.3.1. Role of Nutrition

Individuals with IBD with sub-optimal nutritional status are at risk of poor bone health [86]. Poor nutrition in IBD is multifactorial, including anorexia due to increased disease activity, exclusion diets, medication-induced nausea, painful strictures and malabsorption from both active disease and bowel resections [87]. A significant positive correlation between the body mass index (BMI), an indicator of nutritional status, and DXA-measured BMD Z-scores has been reported [88]. Kherati et al. reported that nearly 63% (*n* = 19/30) of children with CD who were underweight had a BMD Z-score < −2 SD, and a significant correlation between higher BMI and higher BMD Z-scores was also documented [89]. Lower serum albumin (an indicator of malnutrition) has been associated with reduced leg-muscle power in children with CD, which in turn affects bone health [60].

#### 5.3.2. Role of Vitamin D

IBD increases the risk of vitamin D deficiency due to multiple reasons such as impaired fat-soluble vitamin and bile salt absorption, restricted dietary intake, reduced sunlight exposure due to disease restricting activity and on medical advice due to medications such as methotrexate [90,91,92]. Vitamin D deficiency has also been thought to enhance RANKL expression on osteoblasts [93]. A recent metanalysis of 1891 children and adolescents with IBD reported the prevalence of 25 hydroxyvitamin D (25OHD) < 20 ng/mL (50 nmol/L) to be 41% and 25OHD of 20–30 ng/mL (50–75 nmol/L) to be 50% [94]. Vitamin D deficiency and also dietary calcium deficiency can cause secondary hyperparathyroidism, resulting in bone demineralisation and increased fracture risk [95]. Prolonged hyperparathyroidism can lead to phosphaturia, which in turn can result in rachitic changes at the growth plates evident on radiographs [96]. The presence of radiographically confirmed rickets increases the risk of fractures [97].

The typical biochemical signature of vitamin D deficiency includes low serum 25OHD levels, elevated alkaline phosphatase (ALP), elevated parathyroid hormone (PTH) and normal/low serum calcium and phosphate [98]. Information on dietary calcium intake helps assess calcium deficiency [99]. Osteomalacia, reduced mineralisation of pre-formed osteoid, may be diagnosed on typical biochemical features as above but lacks definitive radiological signs [99].

#### 5.3.3. Role of Physical Activity

Regular physical activity during growth is one of the most important factors influencing peak bone mass [100]. There is a positive effect of vigorous physical exercise on bone mineral density [101,102]. Children and adolescents with IBD often feel restricted and demotivated by their condition, resulting in a more sedentary lifestyle [103,104,105]. Werkstetter et al. in a study of 39 children with IBD noted poor grip strength and lesser duration of physical activity compared to controls [105].

## 6. Screening and Monitoring

### 6.1. Clinical Assessment

In children with IBD, regular monitoring of linear growth, growth velocity, pubertal development and menstrual regularity is recommended [106]. A height Z-score < 2 SD (considering parental height potential), faltering height velocity, no secondary sexual characteristics by age 13 years in a girl and 14 years in a boy and no menarche in girls by 15 years of age merit evaluation by a paediatric endocrinologist [106].

**Figure 1:**
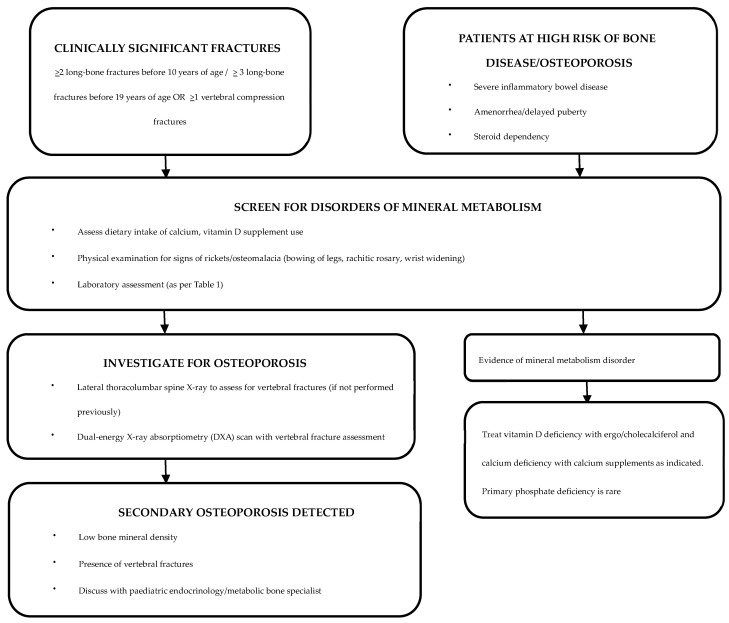

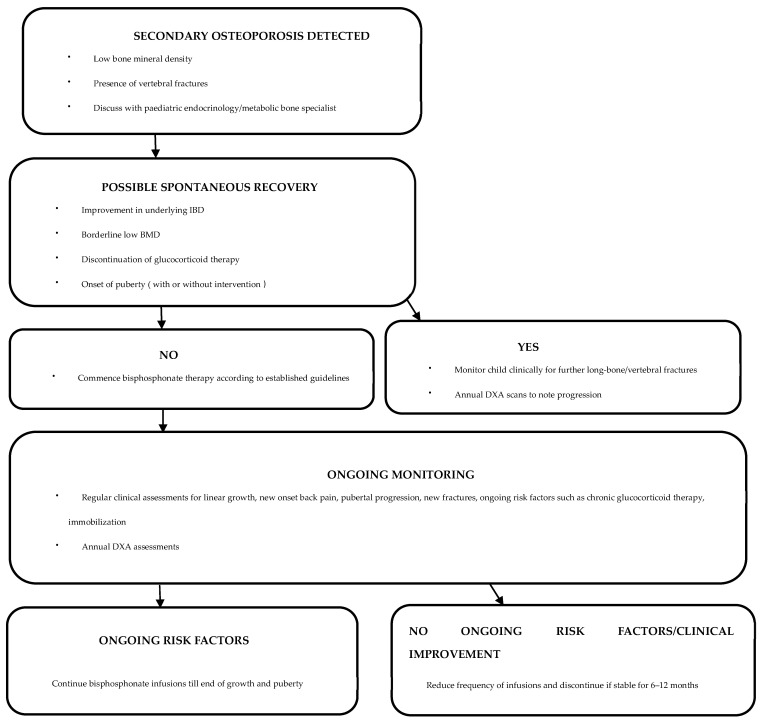
Evaluation and management of patients with IBD who are at risk of bone disease [110].

A history of any clinically significant fractures should be sought during routine consultations, and, if warranted, further evaluation by an endocrinologist or a metabolic bone specialist should be sought [106]. Although most vertebral fractures are asymptomatic, symptoms such as back pain and clinical signs such as spinal tenderness and increase in spinal curvature should be duly noted along with a neurological assessment [107]. As children with IBD are at high risk of vitamin and micronutrient deficiencies, dietary calcium intake and serum 25OHD levels should be assessed at regular intervals [108,109] (Figure 1).

### 6.2. Laboratory Investigations

Recommended screening tests for the identification of underlying disorders of mineral metabolism are detailed in Table 1 and Figure 1.

## 7. Radiological Assessment

### 7.1. Dual-Energy X-Ray Absorptiometry (DXA) Scan

A DXA scan is the gold standard investigation for diagnosis and monitoring of osteoporosis [113]. An annual DXA scan is the preferred screening tool for children and adolescents with IBD who are at risk of osteoporosis [52]. The 2019 International Society for Clinical Densitometry Official Position (Paediatric) statement for skeletal health assessment in children recommend DXA assessment in diseases affecting bone health when the child may benefit from interventions to reduce clinically significant fracture risk [20]. DXA scans are also advised for monitoring bone density while on IBD treatment and if on bisphosphonate therapy (Figure 1) [114]. The European Crohn’s and Colitis Organization (ECCO) and the Paediatric IBD Porto group of European Society of Paediatric Gastroenterology, Hepatology and Nutrition (ESPGHAN) recommend DXA scans to be considered in high-risk patients, such as those with severe disease, prolonged malnutrition, amenorrhea, delayed puberty and/or steroid dependency [115].

### 7.2. Vertebral Imaging

Lateral spine X-ray is a commonly used tool for detection of vertebral fractures [116]. Vertebral fracture assessment (VFA) using DXA is a practical and reliable method to identify clinically relevant vertebral fractures with substantially lower radiation compared to spinal radiography [117]. Whilst magnetic resonance imaging (MRI) is not recommended to diagnose vertebral fractures, if the patient is undergoing imaging for other reasons, the fractures can be identified on MRI scans [118].

## 8. Treatment

### 8.1. General Measures

#### Nutritional Optimisation

Optimisation of nutritional status in childhood may prevent fractures later in adult life [119]. Exclusive enteral nutrition (EEN) has been shown to improve bone metabolism and induce clinical remission in newly diagnosed CD [120]. EEN has been shown to increase bone formation markers along with a reduction in markers of bone resorption [120]. Long-term benefits on bone mass, however, are less well established [121].

### 8.2. Physical Activity

Physical activity has been shown to have a positive impact on quality of life as well as BMD in patients with IBD [122,123]. Trivic et al. in a prospective cross-sectional study of 42 children with IBD demonstrated a strong positive correlation between moderate-to-vigorous physical activity, lean bone mass and BMD [124].

### 8.3. Calcium and Vitamin D Supplementation

The role of calcium supplementation for improving bone health in children with IBD is limited [125]. Benchimol et al. conducted an open-label prospective study, which failed to demonstrate an improvement in BMD in children with IBD who took calcium and vitamin D supplementation [125]. It is, however, worth noting that the study participants had adequate dietary nutrients at the start of the study and were not deficient in calcium or vitamin D [125]. Individuals with IBD are at a higher risk of vitamin D deficiency, and therefore these levels should be kept in check [126]. 25OHD should be monitored at least 6 monthly and supplementation initiated if levels are less than 50 nmol/l [126]. For treatment of vitamin D deficiency in children with malabsorption syndrome such as IBD, the Endocrine Society recommends two or three times the normal doses (6000–10,000 IU per day) for 6–8 weeks, followed by a maintenance dose of 3000 to 6000 IU daily [127]. Children with malabsorption may benefit from intramuscular treatment [128]. In a study including 11 children, Yu et al. demonstrated that 50,000 IU intramuscular weekly was effective and safe in patients with vitamin D deficiency caused by intestinal malabsorption [128]. The recommended daily intake for calcium is 700 mg/day in 1–3 year-olds, 1000 mg/day in 4–8 year-olds, 1300 mg/day in 9–18 year-olds [129].

### 8.4. Therapeutic Modalities

After establishing a diagnosis of secondary osteoporosis due to IBD, it is crucial to determine the possibility of spontaneous recovery in order to establish the need for therapeutic interventions (Figure 1) [110]. Cessation of glucocorticoid therapy and improvement in underlying IBD are likely to increase the chances of spontaneous recovery [110]. Clinical monitoring with annual DXA assessments can be undertaken to assess the progression of osteoporosis [110]. In patients with delayed puberty, pubertal induction contributes towards improving BMD [130]. Children with ongoing risk factors and limited potential for spontaneous recovery are candidates for bisphosphonate therapy [110]. Bisphosphonates should be administered under the supervision of a paediatric bone-health expert [52].

### 8.5. Bisphosphonate Therapy

Bisphosphonates are pyrophosphate derivates that increase bone mineral density by inhibiting osteoclast action, hence reducing bone resorption [131]. Bisphosphonates selectively target bone resorption and not bone formation, thereby improving the cortical width of bones [132]. They exhibit very slow elimination from bone tissue and have been demonstrated to persist in the body for many years after treatment [133]. They are excreted in urine, and are hence contraindicated in renal failure, and dosages must be adjusted to the glomerular filtration rate [134].

Bisphosphonate therapy is effective in improving low bone mineral density and reducing vertebral fracture risk [135]. As expected, bisphosphonates have been shown to increase lumbar spine BMD (LSBMD) in paediatric patients with CD [136]. A metanalysis including nine randomised control trials (*n* = 429) studying bisphosphonate use in secondary childhood osteoporosis demonstrated improvement in LSBMD Z-scores over 3–24 months of follow-up with no increase in the risk of adverse events [137]. Children with vertebral fractures and/or low BMD with clinically significant long-bone fractures should be considered for intravenous bisphosphonate therapy [138]. Prophylactic bisphosphonate therapy in patients with a low BMD Z-score in the absence of fractures is not recommended [138]. As mentioned earlier, BMAD may be normal in the setting of abnormal BMD [23]. In such patients, optimising disease control, nutrition, supplementation and addressing any pubertal delay should be prioritised [138].

IV bisphosphonates (e.g., zoledronate, pamidronate) are more efficacious than their oral counterparts (e.g., alendronate, risedronate) [139,140]. Zoledronate, being 100 times more potent than pamidronate, requires a lower dose and less frequent dosing, making it a more cost-efficient and convenient option for patients [141,142]. The recommended maximum annual dose of IV pamidronate is 9 mg/kg (starting dose 0.5 mg/kg/dose) and IV zoledronate is 0.1 mg/kg (starting dose 0.0125 mg/kg/dose or 0.025 mg/kg/dose) [110,138,142]. In younger children, in view of higher bone turnover, more frequent dosing is recommended [138,142] (Table 2). In older children, at treatment initiation, the annual pamidronate dose can be given in four–six divided doses, while zoledronate can be given in two divided doses per year [138,142].

An acute phase response within 24–48 h (flu-like symptoms) is common and usually resolves with analgesia and hydration [143]. Hypocalcaemia (up to 30% may be symptomatic) may occur after infusion, and the risk can be mitigated by reducing the initial dose and calcium supplementation for 5–10 days after infusions [144,145].

Treatment effectiveness can be monitored through annual DXA assessments [110,138,142]. Bisphosphonate dose and frequency can be reduced or even discontinued following improvement in BMD Z-scores and reshaping of any vertebral fractures [110,138,142]. Occurrence of any new long-bone/vertebral fractures or ongoing low bone density warrants treatment continuation [110,138,142].

### 8.6. Role of Biologics

Elevated TNF-α plays an essential role in cytokine-mediated deterioration in bone health [146]. Infliximab, a TNF-α antagonist used in the medical management of IBD, contributes towards bone formation and decreased bone resorption at the cellular level [147]. Infliximab treatment leads to significant improvement in bone-formation markers [148]. Infliximab therapy has been shown to improve growth, especially in glucocorticoid naïve children and those in the early stages of puberty [149]. Recent studies in adults with IBD have shown beneficial effects of infliximab on BMD [150,151]. Paganelli et al. in a study of 56 patients with IBD, where 10 patients were treated with infliximab, demonstrated that patients who had never received infliximab had significantly lower BMAD than those receiving biologic therapy [152].

### 8.7. Role of Denosumab

Denosumab, a monoclonal antibody against RANKL, is an emerging treatment for bone disorders in children and has been trialled in children with primary and secondary osteoporosis and RANK ligand-mediated disorders [153,154,155,156]. Its widespread utility in children is currently limited by marked suppression of bone turnover with treatment and rebound hypercalcaemia on treatment cessation [157,158]. Its use in paediatric IBD has not yet been evaluated [119].

### 8.8. Role of Growth Hormone

There is no role for growth hormone treatment in IBD except in children with proven growth hormone deficiency [14]. In a prospective study of eight children with CD, 24 months of treatment with recombinant growth hormone did not lead to an improvement in BMD and lean mass, despite improvement in linear growth and bone markers (P1NP) [159].

## 9. Conclusions

Bone health in children with IBD is negatively affected due to multifactorial aetiologies, including poor nutrition, underlying inflammation, delayed puberty and/or prolonged glucocorticoid therapy. Clinicians caring for children and adolescents with IBD should be mindful of the risk factors for poor bone health in this cohort and initiate timely screening. Simple measures such as nutritional supplementation, where indicated, can optimise bone health. Assessment of bone health with DXA scans and prompt initiation of treatment with bisphosphonates, where indicated, can have the potential to improve bone health and prevent long-term adverse effects.

## Figures and Tables

**Table 1:** Laboratory assessment in a child with IBD suspected to have bone disease.

Laboratory Test	Variables
Blood chemistry	Calcium, adjusted calcium *, phosphate, magnesium, alkaline phosphatase, total proteins, creatinine, urea, glucose, 25-hydroxyvitamin D, parathyroid hormone, thyroid-stimulating hormone, free thyroxine
Urine chemistry	Spot calcium, phosphate, creatinine (24 h sampling may be required for further evaluation)
Urine screening	Calcium–creatinine ratio

* Around 50% of the calcium ions are bound to albumin, hence total calcium levels may be falsely low in hypoalbuminaemia [111,112]. Total calcium should therefore be adjusted for albumin levels [111].

**Table 2:** Suggested dosing and frequency for IV bisphosphonates [110,142].

Age	IV Pamidronate (Starting Dose 0.5 mg/kg/Dose, Maximum Dose 9 mg/Year)	IV Zoledronate (Starting Dose 0.0125–0.025 mg/kg/Dose, Maximum Dose 0.1 mg/kg/Year)
<2 years	2-monthly	3-monthly
2–3 years	3-monthly	6-monthly
>3 years	4-monthly	6-monthly
